# Neuroendocrine carcinoma arising in soft tissue: three case reports and literature review

**DOI:** 10.1186/1477-7819-5-77

**Published:** 2007-07-09

**Authors:** Steve L Hyer, Jonathan McAleese, Clive L Harmer

**Affiliations:** 1 Sarcoma Unit, Royal Marsden Hospital, Fulham Road, London, SW3 6JJ, UK

## Abstract

**Background:**

Neuroendocrine tumours (NET) are tumours arising from neuroendocrine cells of neural crest origin. They are characterised by the presence of neurosecretory granules which react positively to silver stains and to specific markers including neuron specific enolase, synaptophysin and chromogranin. Metastasis to the skin occurs infrequently but primary soft tissue NET is excessively rare.

**Case presentation:**

We report our experience with 3 such cases. In the first case, the NET originated in muscle and was treated with wide surgical excision and adjuvant radiotherapy. The second case presented as a subcutaneous mass in the foot and the tumour was positive on ^123^I mIBG scan. She has had prolonged recurrence-free survival following primary hypo-fractionated radiotherapy. In the third case, a cutaneous nodule proved to be a NET and at surgery, lymph node disease was present. He has remained disease-free after surgical excision without the need for external beam radiotherapy.

**Conclusion:**

These tumours appear to have a good prognosis. Complete excision offers potentially curative treatment. Adjuvant radiotherapy may be helpful when the tumour margin is narrow. For patients with unresectable disease or where surgery would not be appropriate, radiotherapy appears to be an effective therapeutic option.

## Background

Neuroendocrine tumours (NET) most commonly originate in the appendix, small intestine, rectum and bronchus [1,2]. The majority are of low-grade malignant potential with an indolent course and can be termed "carcinoids". The release of 5-HT and other vasoactive substances into the systemic circulation gives rise to the "carcinoid syndrome", consisting of flushing, wheezing, diarrhoea and eventually right-sided valvular heart disease [3]. This syndrome is usually associated with bulky liver metastases. Metastasis to skin has been reported but typically occurs in association with metastases elsewhere [4,5]. NET arising *de novo *in the skin are very rare and there are no reports of these tumours originating in soft tissues. We describe our experience of three cases.

## Case presentation

### Case 1

**Figure 1:**
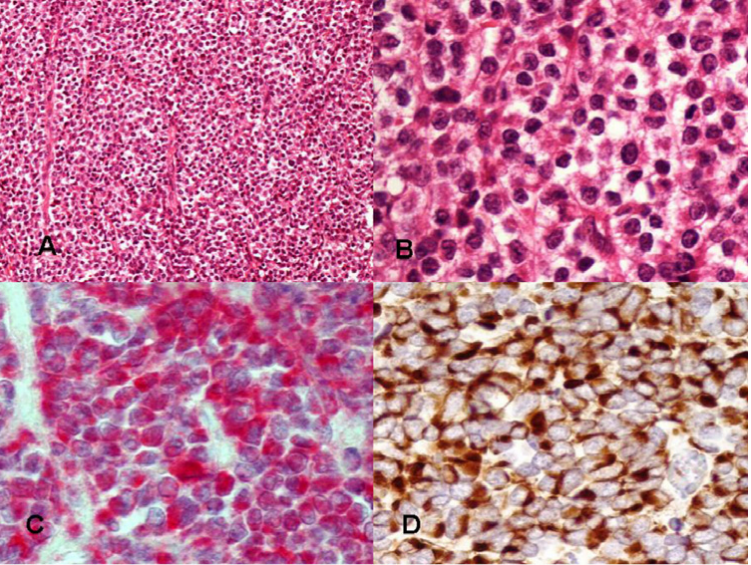
(a) Rows of cells divided by fine fibrovascular stroma into a packet arrangement. This arrangement is typical of an endocrine tumour (H&E × 10). (b) Fine stroma with relatively uniform-looking cells and nuclear irregularity. The nuclear irregularity suggests that the tumour is not benign (H&E × 40). (c) High power showing granular cytoplasmic staining with anti-chromogranin antibody. (d) High power showing diffuse cytoplasmic staining with anti-cytokeratin antibody

A 43 year old man presented with a four month history of a slowly growing hard mass in his right upper thigh. He had no systemic symptoms such as flushing, diarrhoea or wheeze. A 3 × 3 cm mass was excised and found to have arisen in the right sartorius muscle extending into the rectus femoris and vastus medialis. Histopathology revealed skeletal muscle and subcutaneous tissues infiltrated by polygonal cells containing neurosecretory granules (Figures 1a and 1b). Immunohistochemistry showed strong and diffuse positivity for synaptophysin, chromogranin (Figure 1c), cytokeratin (Figure 1d) Bcl-2 protein and neuron-specific enolase (NSE) but negativity for S100, desmin and epithelial membrane antigen (EMA). Electron microscopy showed polygonal cells with deeply indented nuclei and neurosecretory granules in the cytoplasm. These appearances were consistent with a neuroendocrine carcinoma. 24 hour urine 5HIAA concentration was elevated at 92 mmol (normal: 9–31 nmol/24 h).

No tumour was demonstrated elsewhere: mIBG and octreotide scans were negative as were CT scans of thorax, abdomen and pelvis.

Following excision he was treated with adjuvant radical dose external beam radiotherapy to the thigh in view of the depth of invasion of the tumour and its poor histological features. Following treatment his 5HIAA concentrations normalised and he remains free of relapse, five years later.

### Case 2

A 79 year frail old lady presented with a lump on the sole of the right foot which had gradually increased in size over the previous nine months. The right leg had gradually become more swollen and painful. She had not experienced flushing attacks or diarrhoea. On examination there was a hard subcutaneous mass on the plantar surface of the right foot involving the metatarsal heads with associated erythema and thinning of the skin. The entire right leg was swollen with lymphoedema.

**Figure 2:**
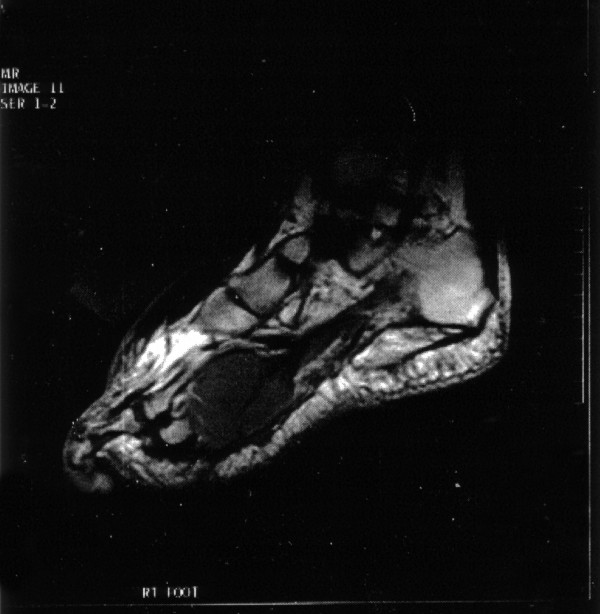
MRI of the right foot in Case 2. A 7 × 5 cm mass is present involving the skin and flexor tendons.

A magnetic resonance (MR) scan was performed of the right foot (Figure 2). This revealed a 7 × 5 cm mass involving skin, flexor compartment of the right sole and flexor tendons. Core biopsy was performed for tissue diagnosis. The appearance was of a necrotic tumour with pleomorphic epithelioid cells. Immunohistochemical staining showed that the cells expressed neuroendocrine markers (synaptophysin, chromogranin, NSE) and cytokeratin marker (CAM 5.2) but were negative for S100, HMB45, CD99 and CD117. The appearances were consistent with a neuroendocrine tumour.

**Figure 3:**
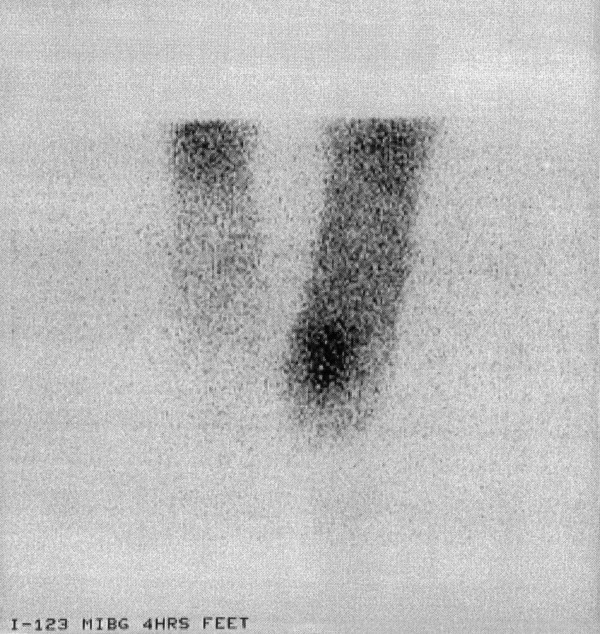
4 hour ^123^I mIBG scan of both feet in Case 2. There is increased uptake of radionucleotide corresponding to the lesion on MRI.

A ^123^I mIBG (meta-iodobenzylguanidine) scan revealed increased uptake into the tumour consistent with its neuroendocrine origin (Figure 3).

CT scans of the chest, abdomen and pelvis showed metastatic lymph node enlargement in the right groin and pelvic side wall but no pathology in the liver or lungs.

Radical surgery was not possible because of the extent of tumour and the patient's general condition. Uptake of mIBG was deemed inadequate to contemplate curative treatment with a therapeutic dose of ^131^I mIBG. Instead, the patient was treated with external beam radiotherapy to the right foot (using 20 MeV electron beam) and to the right side of the pelvis (with parallel pairs of photons). The tumour dose was 30 Gy in 5 fractions with treatment given over 4 weeks (6 Gy once weekly).

MR scanning 10 months later revealed significant reduction in tumour size (3 × 1.5 cm) and no residual pelvic side-wall or inguinal disease. When last reviewed she was well with no cancer-related symptoms. The nodule on the sole of the right foot had not changed over 55 months follow-up and the lymphoedema had completely resolved.

### Case 3

A 61 year old retired civil servant was noted to have a swelling over the left iliac crest. Fourteen months previously he had undergone wide local excision and local radiotherapy for a high grade spindle cell sarcoma affecting the lower left leg. The site was well-healed with no evidence of recurrence. At follow-up, a 2 cm cutaneous nodule was noted over the left iliac crest; it was fleshy and smooth in consistency, being darker than surrounding skin. There were no palpable lymph nodes. He had no systemic symptoms such as flushing or diarrhoea.

Fine needle aspiration cytology (FNAC) of the nodule suggested malignancy. A skin ellipse was resected and included tumour nodules in the deep dermis. Immunohistochemistry showed strong and diffuse positive staining for neuroendocrine markers (NSE, chromogranin, synaptophysin) and CAM 5.2 but was negative for EMA, S100 and HMB45.

Histology was compared with the previous sarcoma but was totally different. Appearances were interpreted as consistent with a neuroendocrine tumour. 24 h urinary 5HIAA concentration was normal. CT scans of thorax, abdomen and pelvis revealed a 1.5 cm inguinal lymph node. FNAC of the node revealed malignant cells. An octreotide scan showed no sign of visceral metastasis or occult primary tumour. A left inguinal dissection was performed; eleven nodes were removed with one showing neuroendocrine tumour differentiation. Surgery was thought to have been complete such that adjuvant radiotherapy was not required. He remains free of recurrence at 79 months follow-up.

## Discussion

Skin or soft tissue involvement in NET is rare and may represent either primary cutaneous disease or metastatic spread from primary neuroendocrine disease elsewhere usually in the lung or gastrointestinal tract [6]. There have been seven previously published reports of primary NET arising *de novo *in the skin but none where the tumour originates in the limbs or soft tissues [7-12]. Previously reported cases of primary skin NET were on the trunk or scalp and were single nodules ranging from 1–4 cm in diameter. The prognosis of these tumours appears favourable as recurrences are rare after prolonged follow-up (Table 1).

**Table 1:** Primary cutaneous neuroendocrine tumours

**Reference**	**Age M/F**	**Clinical**	**Ultrastructure**	**Treatment**	**Course**
Van Dijk C et al, 1975 [7]	72 yr Female	Cystic lesion on forehead with flushing	Argophyllic granules	Excision	Flushing resolved. No recurrence × 1 yr
Brody HJ et al, 1979 [8]	75 yr Female	Hyperpigmented nodule with periumbilical induration. Elevated 5HIAA. Associated hyperparathyroidism	Strongly positive argentaffin reaction	Excision	Not given in report
*Collina et al*, 1988 [26]	80 yr Male	Nodule behind right ear × 12 m	Argophyllic granules. Chromgranin positive Negative argentaffin reaction	Wide excision	No recurrence 21 months later
Bart RS et al, 1990 [9]	40 yr Male	Red-tan soft nodule on abdomen × 4 years	Argophyllic large and varied granules. NSE, keratin +ve	Wide excision	Alive and well 2 years later
Smith PA et al, 1995 [10]	62 yr Female	Firm blue-ish nodule on chest	Argophyllic granules. Postive argentaffin reaction	Wide excision	Alive at 4 years. Well apart from claudication
Courville P et al, 2000 [11]	60 yr Male	Erythematous nodule on chest	Argophyllic granules. Positive argentaffin reaction. Chromogranin A, NSE	Excision	Alive without recurrence 4 years after excision
MacKenzie DN et al, 2003 [12]	70 yr Female	Hard node on scalp with metastasis to occipital lymph node	Synaptophysin, NSE, keratin, CEA +ve	Excision of lesion and occipital lymph node	4 years later further lymph node metastasis. Metastatic node recurrence 2 years later

The diagnosis of primary cutaneous or soft tissue NET depends on exclusion of metastases from an occult tumour elsewhere. Unlike in primary skin NET, skin metastases from an occult primary are usually multiple [9]. Since these tumours are slow growing indolent tumours, metastasis tends to be a late manifestation of advanced disease. The primary occult tumour will usually have attained a diameter of at least 1 cm and not infrequently 2 cm particularly when arising in the appendix or rectum [13,14]. Consequently, the primary tumour is usually large enough for detection by diagnostic imaging. Furthermore, skin metastases from pulmonary or laryngeal NET are usually preceded by symptoms from the primary tumour [15].

Identifying small primary tumours may be difficult with conventional imaging techniques such as ultrasound, CT and MRI [16]. Imaging with ^123^I mIBG shows a sensitivity and specificity of approximately 60% in identifying neuroendocrine tumours [17]. Since NET usually express somatostatin receptors, scanning with radiolabelled somatostatin analogue (octreotide) is often helpful. Compared with mIBG imaging, octreotide scanning is more sensitive (72–87%) although less specific [18].

The presence of metastases is important prognostically as survival is reduced from 70% at 5 years (without metastases) to less than 50% (with metastases) [2].

Macroscopically, a NET usually appears as a yellow solid tumour because of its high lipid content. Histologically, they are composed of rows of cuboidal eosinophilic cells arranged in trabecular, follicular or glandular patterns. Definitive diagnosis requires specific histochemical and immunohistochemical staining. Markers of neuroendocrine differentiation include neuron-specific enolase, synaptophysin and chromogranin. Keratin staining (including CAM 5.2) is generally concentrated at one edge of the nucleus to give an appearance of "dot-like" keratin positivity. Neurosecretory granules can be seen ultrastructurally.

The differential diagnosis includes primary cutaneous neoplasms arising from neurosecretory cells (Merkel cells) in the skin. These tumours, known as Merkel cell carcinomas (MCC), are composed of uniform cells with scanty neoplasm, round to ovoid nuclei with finely dispersed chromatin, inconspicuous nucleoli and frequent mitotic figures. Small membrane-bound dense granules are seen in MCC at electron microscopy in contrast to NET where granules are often large and irregular. Early reports of neuroendocrine differentiation in MCC utilised silver staining reactions and demonstrated argyrophilia but negative argentaffin reactions [19]. These have largely been superseded by specific stains for neuroendocrine markers such as chromogranin which can be positive in either MCC or NET. Immunohistochemical stains in MCC tumours show variable positivity for cytokeratin and CK20 often in a perinuclear arrangement in contrast to the more diffuse staining seen in NET [20] and in the cases reported here. More recently, distinctive cytogenetic abnormalities have been described in MCC which may help differentiate MCC from NET [21].

Clinically, MCC tumours are characterised by aggressive behaviour with local recurrences, frequent metastases and relatively poor response to conventional therapies [21,22]. In case 1 the tumour clearly arose from muscle. The tumour in case 2 could have originated in the skin and invaded deeply as the histology showed it to be in the subcutaneous tissue. However, it would have been MCC stage II as the lymph nodes were involved and these tumours have a median survival of only 5 months [23]. Similarly, the long recurrence-free survival in case 3 (also with lymph node disease) makes this unlikely to have been a MCC.

As with previous reports of cutaneous NET, primary surgical excision was the preferred treatment in our cases, combined with nodal clearance in (case 3) or adjuvant radiotherapy (case 1). Excision of a skin NET and lymph node metastasis was successful in a previous case report [12]. Disease control in our patients has been good at long term follow-up.

In case 2, primary hypofractionated radiotherapy was used as radical surgery was not possible. This approach has been used for several other tumours e.g bladder, prostate where patients are frail and cannot tolerate conventionally fractionated radical doses [24]. Radiotherapy has been used successfully to control unresectable NET in the brain, spinal cord and bone [25]. Disease regression and symptom palliation was achieved in the majority of patients.

We believe these cases represent primary NET because no evidence of tumour could be found elsewhere after follow-up of 5 years. In only one patient were urinary 5 H-T metabolites increased and this patient did not experience features of the carcinoid syndrome presumably because the quantity of peptides secreted was too low.

The soft tissue lesions in all our patients had advanced slowly over a period of between 4 to 9 months and had metastasized only to regional lymph nodes and not to distant organs.

In summary, these tumours appear to have a good prognosis. Complete excision offers potentially curative treatment. Staging is required to exclude lymph node or distant metastases. Adjuvant radiotherapy may be helpful when the tumour margin is narrow. Patients with recurrent disease or aggressive histopathological appearances require radical radiotherapy doses. Although potentially beneficial, uptake of mIBG and octreotide is probably insufficient in the majority of cases to deliver tumouricidal doses. For patients with unresectable disease or where surgery would not be appropriate, radiotherapy appears to be an effective therapeutic option.

## Competing interests

The author(s) declare that they have no competing interests.

## Authors' contributions

SLH preparation of final draft, literature search, corrections and response to reviewers

JM preparation of initial draft, collation of patient information

CLH idea for the study, identification of patients, manuscript preparation and revisions

All authors read and approved final manuscript.
